# Cell surface GRP78 as a biomarker and target for suppressing glioma cells

**DOI:** 10.1038/srep34922

**Published:** 2016-10-07

**Authors:** Bo Ram Kang, Seung-Hoon Yang, Bo-Ryehn Chung, Woong Kim, YoungSoo Kim

**Affiliations:** 1 Convergence Research Center for Dementia, Brain Science Institute, Korea Institute of Science and Technology, Seoul, Republic of Korea; 2 Biological Chemistry Program, Korea University of Science and Technology, Daejeon, Republic of Korea; 3 Department of Pharmaceutical Science, College of Pharmacy, Kyung Hee University, Seoul, Republic of Korea; 4 Department of Neuroscience, Princeton University, New Jersey, USA; 5 Third Department of Internal Medicine, School of Medicine, Wakayama Medical University, Wakayama, Japan

## Abstract

High-grade glioma is a highly malignant and metastatic brain cancer, resistant to many existing anticancer treatments. In such glioma cancer cells, the glucose-regulated protein 78 kDa (GRP78) is particularly highly up-regulated. Former studies have thus targeted mutation-free GRP78 not only to detect glioma cancer cells specifically but also to enhance cytotoxic effect. We focus on cell surface-expressed GRP78 as a target for suppressing high-grade glioma cell lines. Glioblastoma multiforme (GBM) cell line, highly malignant glioma cells, was first injected into 5-week-old athymic mice to confirm and compare GRP78 expression *in vivo* in xenografted and normal brain tissue. Immunofluorescence and immunoblotting were utilized to detect surface-localized GRP78 in diverse high-grade glioma cell lines. By treating glioma cell lines with the polyclonal N-20 antibody against surface-localized GRP78, we subsequently studied the significance of surface GRP78 to the survival and growth of the glioma cell lines. We found that inhibiting the function of surface GRP78 suppressed cancer cell survival and growth proving that the surface-expressed GRP78 is a vital receptor involved in the proliferation of high-grade glioma. Our findings provide opportunities to target surface GRP78 as a biomarker for high-grade glioma and to develop effective cell-specific anticancer therapy.

Despite advances in anticancer therapy, glioma remain difficult to treat. The complex multiforme nature – the genetic heterogeneity and pleomorphic cells – make the tumour difficult to target and highly resistant to current cancer therapies. Such cancer treatments have additionally targeted biomarkers of glioma that are capable of mutating^1^. Thus, suppression of tumour survival and growth through receptor inhibition is a promising method to potentially treat gliomas.

Such invasive characteristics of glioma are known to be regulated by the glucose-regulated protein 78 kDa (GRP78), a member of the heat shock protein 70 family. GRP78 predominantly resides in the endoplasmic reticulum lumen where it plays an important role in proper assembling of proteins and targeting misfolded proteins for proteosomal degradation^2^. However, recent studies have found highly elevated GRP78 expression in tumour microenvironments, resulting in active translocation of GRP78 to the surface of cancer cells^3^. It is on the surface of highly invasive tumour cells where GRP78 has been postulated to assume proliferative roles^4^. Surface-localized GRP78 has been reported to promote tumour survival, invasion and resistance to cell apoptosis in breast, liver, prostate, colon and gastric cancer cell models *in vitro*^5^,^6^,^7^,^8^,^9^. Consequently surface GRP78 is strongly implicated to mediate signal transduction pathways inducing cancer cell viability and metastatic ability.

Our study investigates the effects of inhibition of surface GRP78 in high-grade glioma cell lines. First, to confirm the expression of GRP78 in glioma cell line *in vivo*, we injected cells of U-87 MG human glioblastoma multiforme (GBM) which accounts for 80% of all malignant brain cancer, into 5-week-old athymic mice and compared GRP78 expression in xenografted and normal brain tissue. We then performed an immunoblot to confirm high expression of GRP78 in several select high-grade glioma cell lines. Expression of surface GRP78 in the high-grade glioma cell lines was detected by immunocytochemistry and immunoblotting. To subsequently study the significance of surface GRP78 in tumour survival and growth, we treated the high-grade glioma cell lines with a polyclonal antibody N-20 against surface GRP78, thereby neutralizing its function.

## Results

### GRP78 highly expressed in GBM of xenografted brain mice model

**Figure 1:**
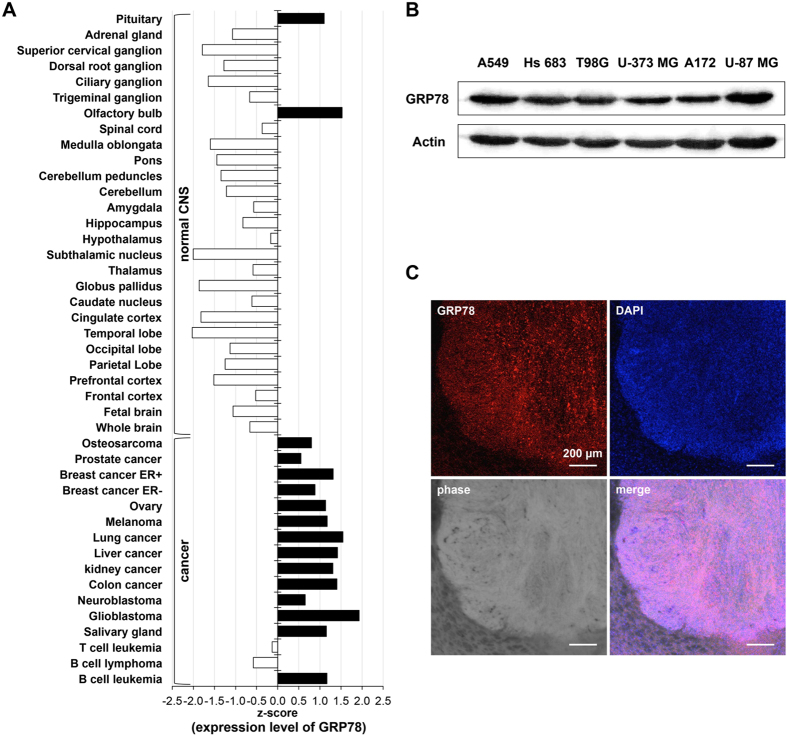
Up-regulated expression of GRP78 in glioma. (**A**) Profile of GRP78 gene expression in tissues of normal central nervous system and cancers. X-axis is marked in standard deviations from the GRP78 expression mean; values of z-score correlate with GRP78 expression. (**B**) Western blot analysis of lysates from glioma cell lines and A549 cell line as positive control for detecting GRP78. Expression of actin served as a loading control. (**C**) Immunohistological analysis for GRP78 expression in the thalamus of xenografted mouse brains after 28 days of U-87 MG cells injection. DAPI used for labeling cell nuclei. Scale bar = 200 μm.

The profile of gene expression shows that among the normal central nervous system (CNS) tissue, brain tissue has the lowest average expression of GRP78. However, among cancer tissues, glioblastoma, a highly malignant and metastatic type of glioma, has the greatest up-regulation expression of GRP78 (Fig. 1A and Supplementary Figure S1). By western blot, we confirmed the expression of GRP78 in the high-grade glioma cell lines utilized in this study. Upon comparison to the human lung adenocarcinoma cell line, A549, which is known to highly express GRP78^10^, the high-grade glioma cell lines (Hs 683, T98G, U-373 MG, A172 and U-87 MG) likewise displayed high expression of GRP78 (Fig. 1B).

To confirm the higher expression of GRP78 in GBM cell line than in normal tissue *in vivo*, we generated xenografted mice by injecting GBM cells (U-87 MG) into the right striatum of 5-week-old athymic mice. We found a high expression of GRP78 in the GBM of the brains of xenografted mice 28 days post-injection (Fig. 1C), indicating that the up-regulation of GRP78 in high-grade glioma cell lines is likewise shown in xenografted animal models.

### GRP78 expressed on the cell surface of high-grade glioma cell lines, and surface-localized GRP78 interaction with neutralizing antibody

**Figure 2:**
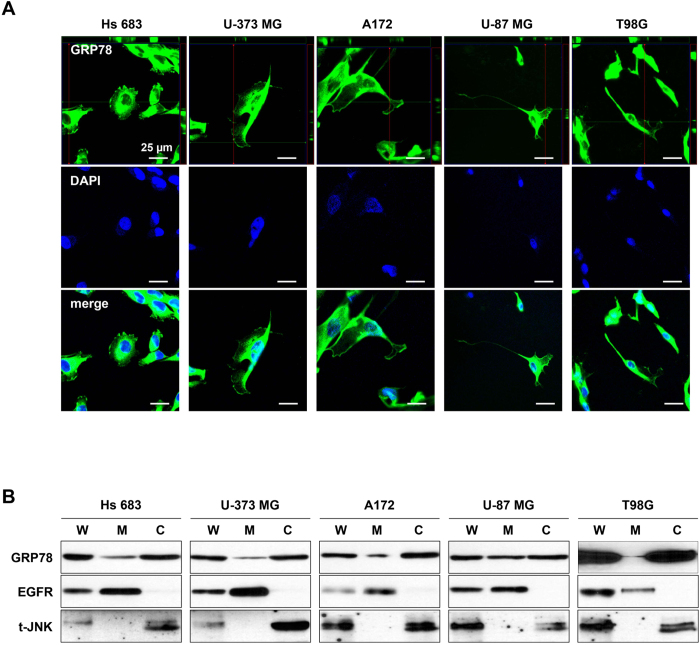
Abnormal localization of GRP78 on surface of glioma cell lines. (**A**) Confocal microscopic analysis for GRP78 expression in high-grade glioma cell lines. DAPI used for labeling cell nuclei. Scale bar = 25 μm. (**B**) Western blot analysis of cytosol and plasma membrane proteins from high grade glioma cell lines to detect GRP78. EGFR and t-JNK indicated the membrane and cytosol proteins respectively. W, whole cell lysates; M, plasma membrane; C, cytosol.

GRP78 of several cancer cells is known to be expressed on the cell surface while normal cells typically express GRP78 only within the cell^2^,^9^. To locate where GRP78 was localized on our high-grade glioma cell lines, we performed an immunocytochemistry assay and immunoblotting using a monoclonal GRP78 antibody. As expected, GRP78 expression appeared not only in the cytosol but also on the surface of A549 cells^10^ (Supplementary Figure S2A,B) and the high-grade glioma cell lines (Fig. 2A). Isolation and immunoblotting analysis of whole cell lysates, cytosol and plasma membrane protein further confirmed the localizations of GRP78 in both the plasma membrane and cytosol of the glioma cell lines (Fig. 2B). The efficiency of plasma membrane and cytosol protein isolation was analysed by detecting epidermal growth factor receptor (EGFR) and total-c-Jun N-terminal kinase (t-JNK) respectively.

**Figure 3:**
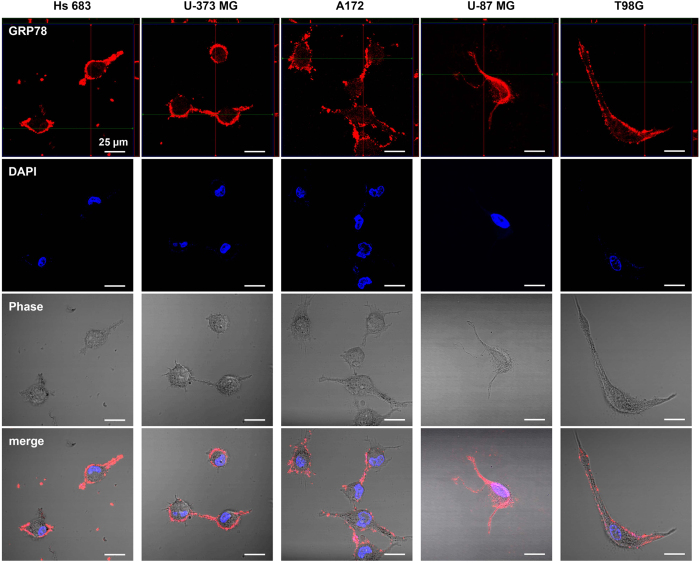
Interaction of N-20 antibody with surface GRP78 of the glioma cell lines. The live cell lines were incubated with the polyclonal N-20 antibody, and the binding of the polyclonal N-20 antibody with surface GRP78 visualized using a fluorescent secondary antibody. DAPI used for labeling cell nuclei.

The polyclonal N-20 antibody against GRP78 is known to neutralize surface GRP78 signalling^6^,^11^,^12^. To examine the effect of the inhibition of surface GRP78 on the survival and growth of high-grade glioma cell lines, we treated the polyclonal N-20 antibody into the cells. Prior to this, we first confirmed the exclusive interaction of polyclonal N-20 antibody with surface GRP78 by staining the high-grade glioma cells with a secondary antibody conjugated to a fluorescent dye. We found that the antibodies were bound to GRP78 located on the cell membranes of all glioma cell lines (Fig. 3). These results imply that surface GRP78 of high-grade glioma has no mutation at the N-terminal, the epitope for the N-20 antibody, and that surface localized GRP78 functions as a receptor.

### Treatment of surface GRP78 with antibody N-20 suppresses high-grade glioma cell line survival and population growth

**Figure 4:**
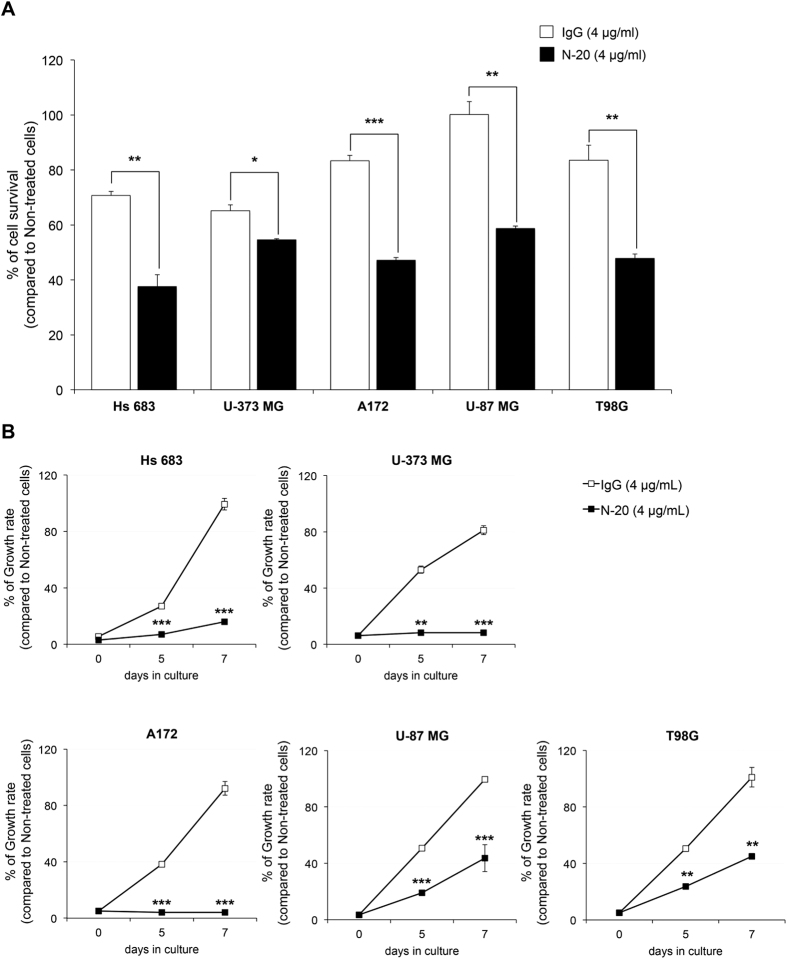
Suppression of cell survival and growth by blocking of surface GRP78. High-grade glioma cell lines were incubated with 4 μg/mL goat isotype IgG or polyclonal N-20 antibody. (**A**) Cell survival was assessed using MTT assay where values show antibody N-20 treatment results compared to IgG treated control. (**B**) Growth curves were monitored with crystal violet staining at indicated time points. Stain was dissolved and the absorbance measured at 560 nm. The results are presented as the mean values ± standard deviation of triplicate determinations. The data were analysed using a student’s *t*-test (*P < 0.05, **P < 0.01, ***P < 0.001). IgG and N-20 indicate goat IgG isotype and polyclonal N-20 antibody treatment, respectively.

To identify whether surface GRP78 expression is involved in cancer cell survival and growth signalling in high-grade glioma cell lines, we treated polyclonal N-20 antibody into the cell lines and measured tumour cell viability by an MTT cytotoxicity assay. The percentage of survival of the treated glioma cell lines markedly decreased compared to that of the goat IgG isotype treated controls (Fig. 4A). We also found almost a complete inhibitory effect on population growth of the human anaplastic glioma (WHO grade III) cell lines, Hs 683 and U-373 MG, and the human GBM (WHO grade IV) cell line A172 upon treatment with the polyclonal N-20 antibody (Fig. 4B). The remaining human GBM cell lines, U-87 MG and T98G, almost halved in population growth rate upon polyclonal N-20 antibody treatment. As expected, we found a greater reduction in growth rate for the lower pathological cell grade; the anaplastic glioma cell lines exhibited higher suppression of cell growth than the GBM cell lines. The rate of population growth of the glioma cell lines 7 days post treatment with the IgG-control did not change. These results suggest that surface GRP78 plays a role of cell proliferation in high-grade glioma.

## Discussion

Normal brain tissues have lower expression of GRP78 than other normal tissues. However, among cancer tissues, glioblastoma has the highest expression of GRP78 (Fig. 1A). It has additionally been reported that GRP78 expression increases with pathologic grade level of glioma^13^. This overexpression of GRP78 in high-grade glioma is considered strongly associated with glioma progression, thus we investigated the up-regulated GRP78 in high-grade gliomas and its significance in tumour survival and growth. We found that GRP78 was abnormally localized on the cell surface of such cells, accounting for the high expression of GRP78 in particularly glioma cells. There was additionally no mutation found on the epitope for the inhibitory antibody (Figs 2 and 3). Upon treatment of polyclonal N-20 antibody into glioma cells that were cultured on a coverglass, we found that the surface GRP78 exclusively interacted with the polyclonal N-20 antibody. This interaction between surface GRP78 and polyclonal N-20 antibody implies that the surface localized GRP78 may function as a receptor involved in glioma proliferative signaling and can thus be suppressed through binding with N-20 antibody. Polyclonal N-20 antibody has been reported to block the receptor function of cell surface GRP78^11^,^12^,^14^. Indeed, inhibition of surface GRP78 by polyclonal N-20 antibody treatment suppressed glioma cell survival and growth, showing great potential for use in cancer-specific therapy (Fig. 4). Thus, our study suggests that surface GRP78 could be a novel target for high-grade glioma treatment.

Our results have promising clinical implications in advancing cancer-specific therapy. GRP78 has been shown to not form mutations and its overexpression is known to be induced by tumour microenvironments^15^. In addition, GRP78 induction has been linked to the development of resistance to various cytotoxic agents^16^,^17^. Thus, GRP78, and in our case surface GRP78, is an attractive target receptor of cancer cells and can act as a site of delivery of anticancer drugs and consequent suppression of cancer viability. Agents inhibiting the synthesis, stability or activity of surface GRP78 can suppress the protein’s function in cancer proliferation^4^ and can be used in conjunction with other standard cytotoxic agents to enhance efficacy of anticancer treatment for glioma and other highly metastatic and resistant cancers exhibiting surface GRP78.

Since glioblastoma, the glioma of the highest tumour grade, has the highest expression of GRP78 among cancers, it is the ideal cell model for studying the role of GRP78 in highly metastatic cancers. However, since our study utilizes cell lines of glioma, the use of *in vivo* or *ex vivo* methods to confirm our findings would strengthen our claim that surface GRP78 is indeed involved in the pro-proliferative and antiapoptotic mechanisms of glioma. Further investigation is also warranted on the mechanisms involving surface GRP78 in order to further understand the role of surface GRP78 in cancer cell proliferation. Moreover, analysing the causes of GRP78 surface expression in cancer cells but not in non-cancer cells can be greatly beneficial for postulating specific cancers that highly express surface GRP78. This paper opens new areas of investigation which would greatly benefit the prognosis of gliomas and other brain tumours strongly expressing GRP78.

## Methods

### Gene expression analysis

To investigate the cell-specific expression level of GRP78 in human tissues, we used an online tool Gene Enrichment Profiler (http://xavierlab2.mgh.harvard.edu/EnrichmentProfiler/index.html). In this database, the expression enrichment of any set of query genes was computed on the basis of a reference set obtained from 126 normal tissues and 16 cancer types represented by 649 microarrays^18^.

### Cell cultures

Human lung adenocarcinoma cell line (A549), human anaplastic glioma cell lines (WHO grade III, Hs 683 and U-373 MG) and human GBM cell lines (WHO grade IV, T98G, A172 and U-87 MG) were purchased from the Korean Cell Line Bank (Seoul National University, Republic of Korea). These cell lines were cultured in Dulbecco’s modified Eagle’s medium (Gibco, USA), supplemented with 10% fetal bovine serum (Gibco, USA) and 1% penicillin-streptomycin antibiotics (Gibco, USA). All cell lines were maintained at 37 °C in a humidified atmosphere of 5% CO_2_ in air.

### Isolation of plasma membrane and cytosol protein

Plasma membrane and cytosol protein were isolated using a membrane protein extraction kit (Abcam, UK). In brief, cells were scraped and washed with cold PBS. Cells were re-suspended and homogenized in an ice-cold dounce homogenizer, then centrifuged at 700 × g for 10 minutes at 4 °C. Supernatants were collected and centrifuged at 10,000 × g for 30 minutes at 4 °C. The supernatants (cytosol) were collected and the pellets as the total cellular membrane protein were re-suspended in upper and lower phase solution. The lysates were centrifuged at 3,500 rpm for 5 minutes with the resulting pellets (plasma membrane) collected.

### Immunoblotting

Glioma cell lines were lysed in RIPA buffer (Sigma, USA), supplemented with competent protease inhibitor cocktail tablet (Roche Applied Science, Germany). Cell lysates were then centrifuged at 15,000 rpm for 20 minutes at 4 °C. The proteins were resolved on 10% sodium dodecyl sulfate polyacrylamide gel, followed by electrotransfer to a nitrocellulose membrane. Target protein was probed with the indicated antibody as follows: mouse monoclonal anti-GRP78 antibody (BD biosciences, USA), rabbit polyclonal anti-EGFR antibody (Santa Cruz Biotechnology, USA) and rabbit polyclonal anti-total JNK antibody (Cell signalling, USA). Immunodetection was carried out using HRP-labelled secondary antibody and then protein levels were detected with an ECL substrate (Bio-Rad, USA).

### Murin xenograft tumour models generation

U-87 MG cells were propagated, collected after trypsinization, and resuspended in ice-chilled PBS. 5-week-old athymic mice (CAnN.Cg-Foxnl^nu^/Crl) were anesthetized by intraperitoneal injection of 15 mg/kg Zoletil 50 (Virbac, France) and 6 mg/kg Rompun (Bayer HealthCare AG, Germany) anesthetic cocktail. A small hole was drilled through the skull, by means of a stereotactic device (David Kopf instruments, USA), at 0.2 mm posterior and 2.2 mm lateral to the bregma. A 26-gauge needle (Hamilton company, USA) was inserted to a depth of 3.5 mm from the skull surface. Cells (1 × 10^5^) were injected into the right striatum of the mice. Mice were maintained in a temperature- and light-controlled environment with an alternating 12 hours light/dark cycle. All animal experiments were carried out in accordance with the National Institutes of Health guide for the care and use of laboratory animals (NIH Publications No. 8023, revised 1978). The animal study protocol was approved by the Institutional Animal Care and Use Committee of Korea Institute of Science and Technology (AP-2011L1015).

### Immunostaining

For the immunohistochemical stain, Mice were sacrificed 28 days after injection. Brains were perfused and fixed by 4% paraformaldehyde. Fresh frozen tissue embedded in optimal cutting temperature compound was sectioned at 50 μm. After blocking with 2% bovine serum albumin (Sigma, USA), the sections were incubated with goat polyclonal anti-GRP78 (N-20) antibody (1:100, Santa Cruz Biotechnology, USA) at 4 °C overnight. Anti-goat IgG H&L (Cy5^®^) secondary antibody (1:200, Abcam, UK) were used for fluorescent detection.

For the immunocytochemical stain, the glioma cell lines were cultured in a glass bottom dish. Cells were fixed with 4% paraformaldehyde and blocked with 1% bovine serum albumin. The cells were incubated with primary mouse monoclonal anti-GRP78 antibody (1:200) at 4 °C overnight. Antibody binding was localized with anti-mouse Alexa Fluor^®^ 488 (1:200, Invitrogen, UK). To present live cell imaging, the glioma cells were treated with goat polyclonal N-20 antibody (1:100) for 1 hour at 37 °C while cells live. After fixation, antibody binding was localized with anti-goat IgG H&L (Cy5^®^) secondary antibody (1:200). Nuclei were counterstained with DAPI. Images were obtained by a Carl Zeiss LSM 700 microscope and analysed by ZEN 2012 software (Carl Zeiss, Germany).

### MTT cytotoxicity assay

The glioma cell lines were plated at 5 × 10^3^ cells/well in a 96 well plate. 24 hours later, cells were incubated for 72 hours with 4 μg/mL of goat polyclonal N-20 antibody or the normal goat IgG (Santa Cruz Biotechnology, USA) as a vehicle control. Cell viability was assessed using a CellTiter 96^®^ Non-Radioactive Cell Proliferation Assay (Promega, USA). Briefly, cells were incubated with 15 μL dye solution for 4 hours, and formazan crystals formed in cells were dissolved by stop solution. Absorbance was measured at 570 nm using an EnSpire plate reader (Perkin-Elmer, USA).

### Growth curve assay

The glioma cell lines were seeded in a 96 well plate at a density of 1 × 10^3^ cells/well and incubated overnight. Then the culture media was changed to 200 μL conditioned media which has 4 μg/mL goat polyclonal N-20 antibody or the control goat isotype IgG (Sigma, USA). These cells were allowed to grow for 7 days total. At 1, 5 and 7 days of incubation, the cells were fixed and stained with crystal violet staining solution for 20 minutes. Stained cells were washed three times with deionized water and allowed to air-dry. Then 10% acetic acid was added to each well to dissolve the stain. Absorbance was measured at 560 nm using an EnSpire plate reader.

## Additional Information

**How to cite this article**: Kang, B. R. *et al*. Cell surface GRP78 as a biomarker and target for suppressing glioma cells. *Sci. Rep.* **6**, 34922; doi: 10.1038/srep34922 (2016).

## Supplementary Material

Supplementary Information
