# Supplementary material for: Cell surface GRP78 as a biomarker and target for suppressing glioma cells
Source: Sci Rep. 2016 Oct 7;6:34922. doi: 10.1038/srep34922 (PMC5054676; doi:10.1038/srep34922)

SUPPLEMENTARY INFORMATION

Cell surface GRP78 as a biomarker and target for suppressing glioma cells

Bo Ram Kang, Seung-Hoon Yang, Bo-Ryehn Chung, Woong Kim & YoungSoo Kim

Supplementary figure legends

Supplementary Figure S1. GRP78 expression level of each human normal primary tissue Profile of GRP78 gene expression in normal tissues of human. The x-axis is marked in standard deviations from the expression mean. The higher value of the z-scores shows more increased expression.

Supplementary Figure S2. Expression of GRP78 in A549 human lung adenocarcinoma cell line (A) Confocal microscopic analysis for GRP78 expression in A549 cell line. DAPI used for labeling cell nuclei. Scale bar = 25 µm. (B) Western blot analysis of cytosol and plasma membrane proteins from A549 cell lines to detect GRP78. EGFR and t-JNK indicated the membrane and cytosol proteins respectively. W, whole cell lysates; M, plasma membrane; C, cytosol.

Supplementary Figure S1

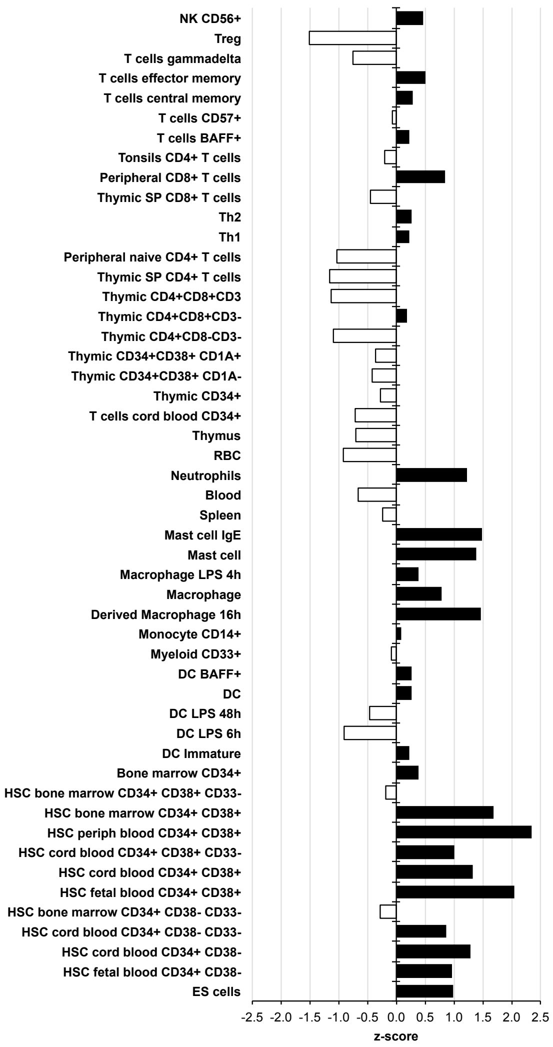

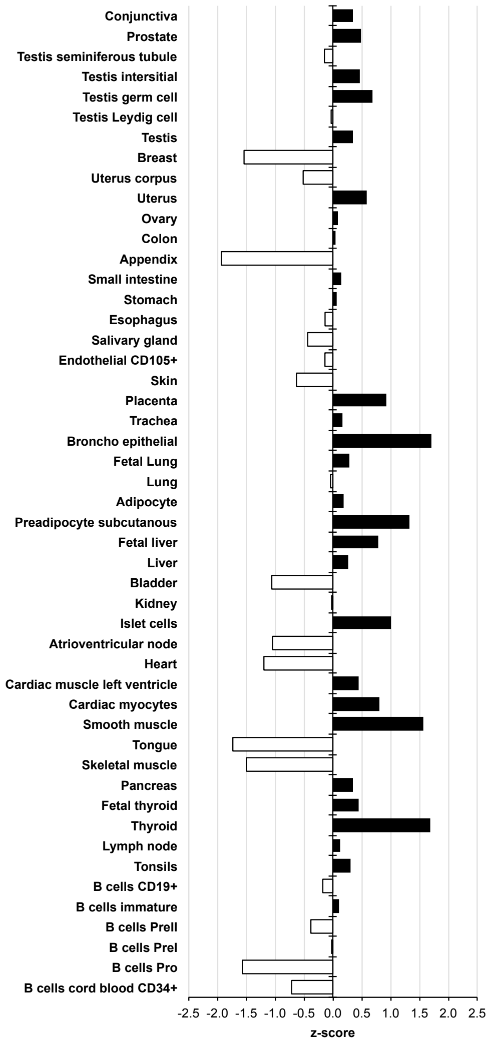

Supplementary Figure S2

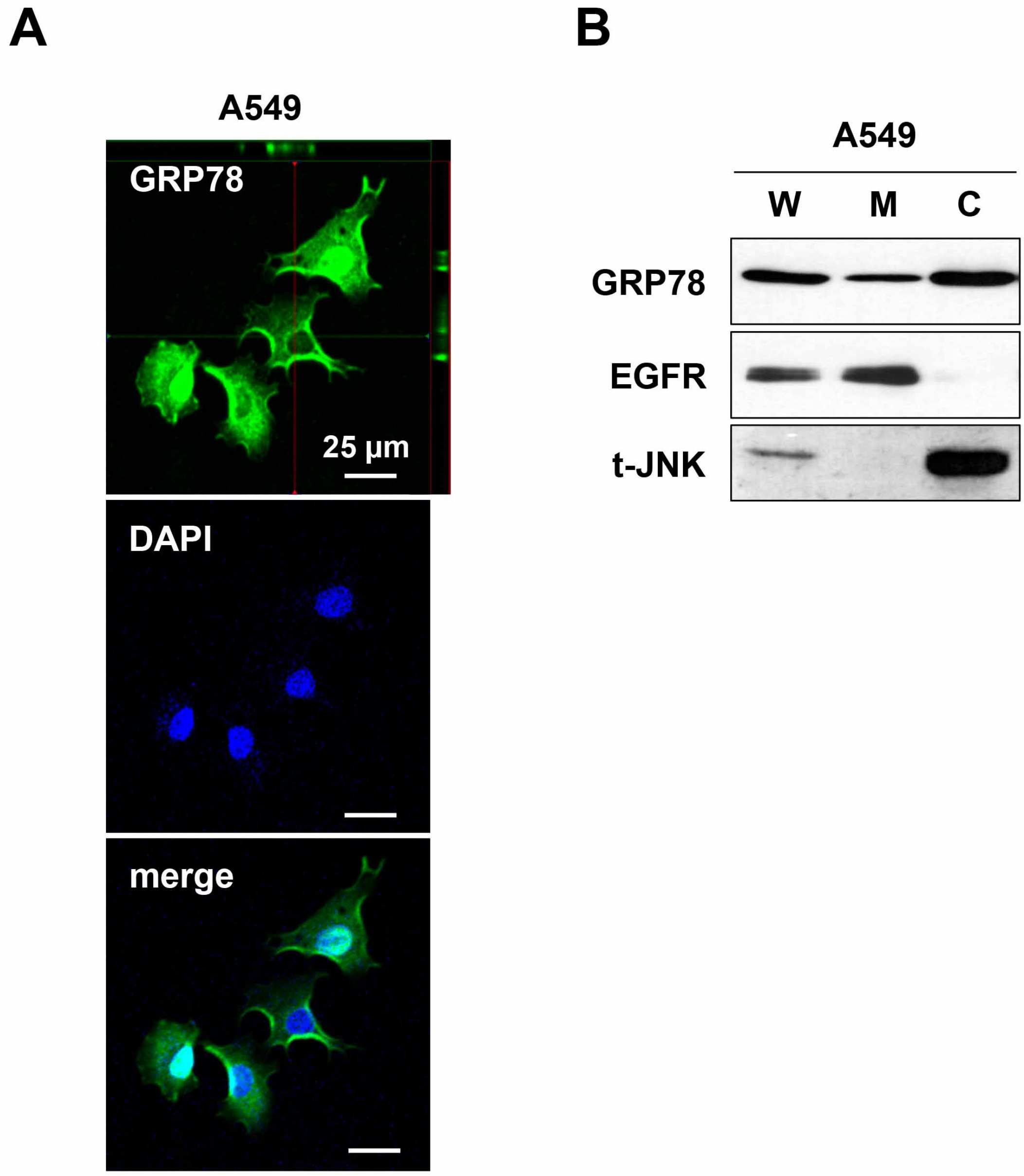

Supplement: Supplementary Information [file srep34922-s1.pdf]
